# Supplementary material for: Assessing European cities with the 3-30-300 rule underscores the need for enhanced urban greening efforts
Source: Nat Commun. 2026 Apr 9;17:4846. doi: 10.1038/s41467-026-71523-8 (PMC13223244; doi:10.1038/s41467-026-71523-8)
Supplement: Supplementary file 1 — Supplementary Information [file 41467_2026_71523_MOESM1_ESM.docx]

Supplementary information for:

**Assessing European Cities with the 3-30-300 rule underscores the need for enhanced urban greening efforts**

L.E. Bertassello ^1*^, M. van der Velde ^1^, J. Maes ^2^, S. Liu ^3^, M. Brandt ^3^, L. Feyen ^1^

1. European Commission, Joint Research Centre (JRC), Ispra, Italy
2. European Commission, Directorate-General for Environment (DG-ENV), Brussels, Belgium
3. Department of Geosciences and Natural Resource Management, University of Copenhagen, Copenhagen, Denmark

* Corresponding Author: Leonardo Enrico Bertassello, [leonardo.bertassello@ec.europa.eu](mailto:leonardo.bertassello@ec.europa.eu)

**List of Supplementary Items**

[**Supplementary Figures** 3](#_Toc224468979)

[**Supplementary Tables** 14](#_Toc224468980)

[**Supplementary References** 16](#_Toc224468981)

# **Supplementary Figures**


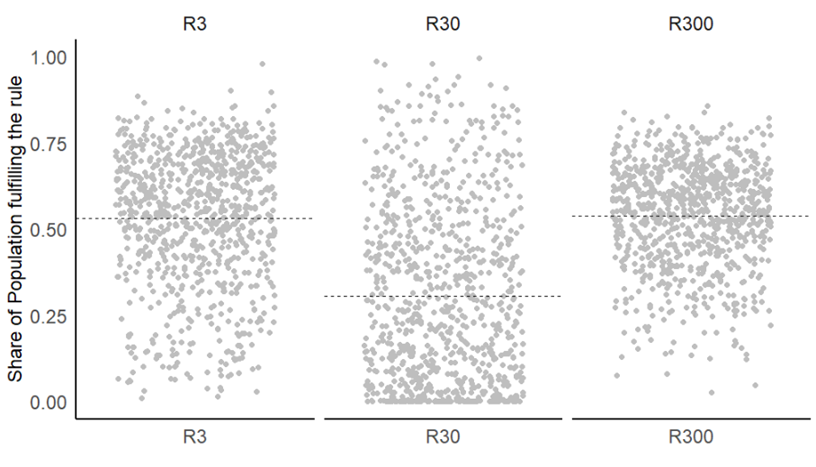


**Supplementary Figure 1:** Proportion of the population meeting each of the three individual criteria of the 3-30-300 rule across 862 cities. The dashed line indicates the average values.


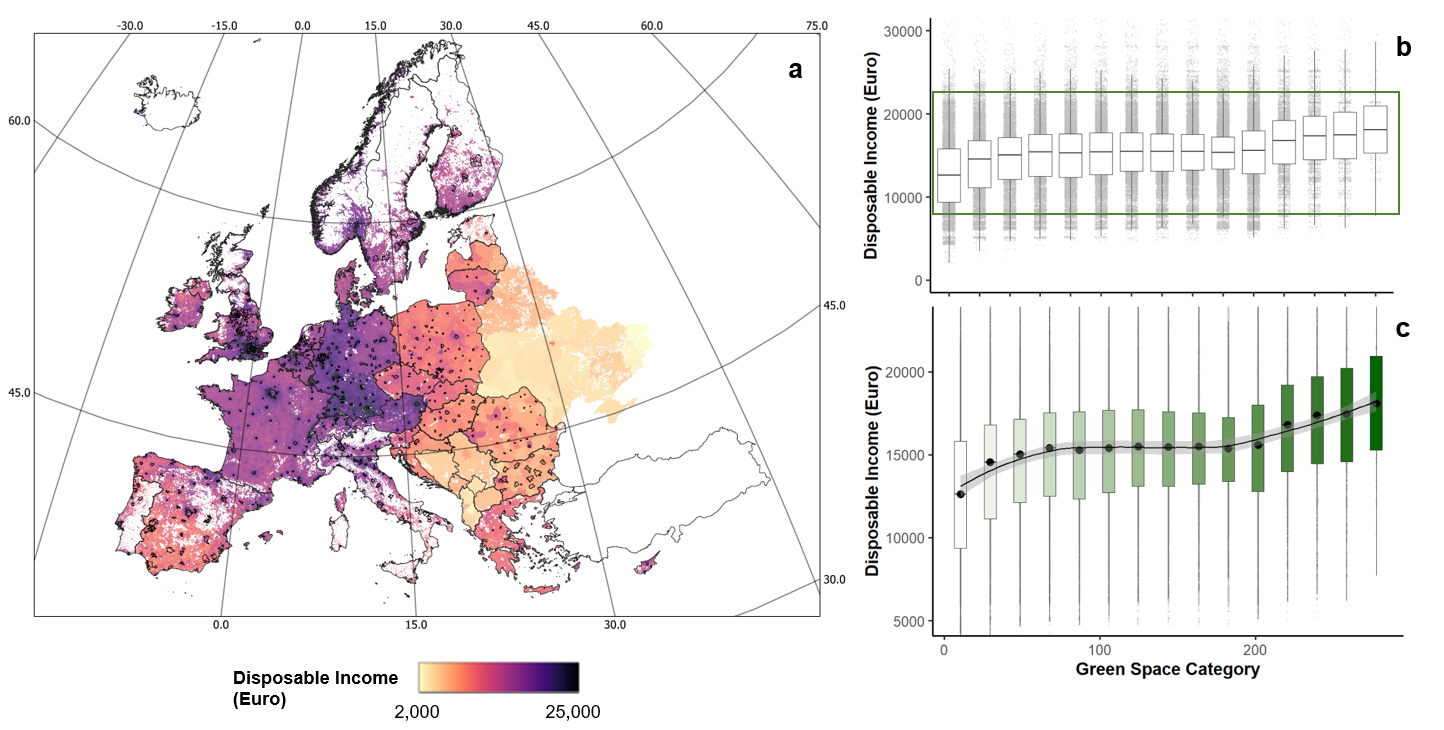


**Supplementary Figure 2:** (a) Disposable income across all EU27 + UK,CH,NO cities obtained from Mikou et al., 2025. at 1x1 km spatial resolution. (b) Values of the Green Space Category in the x-axis are obtained as the average of the discrete pixel values of compliance with the 3-30-300 rules (0, 1, 2 or 3) over the 1 x 1 km grid cells falling in the coarser disposable income grid cell. (c) Zoom for the blue rectangle presented in of Figure S2b.


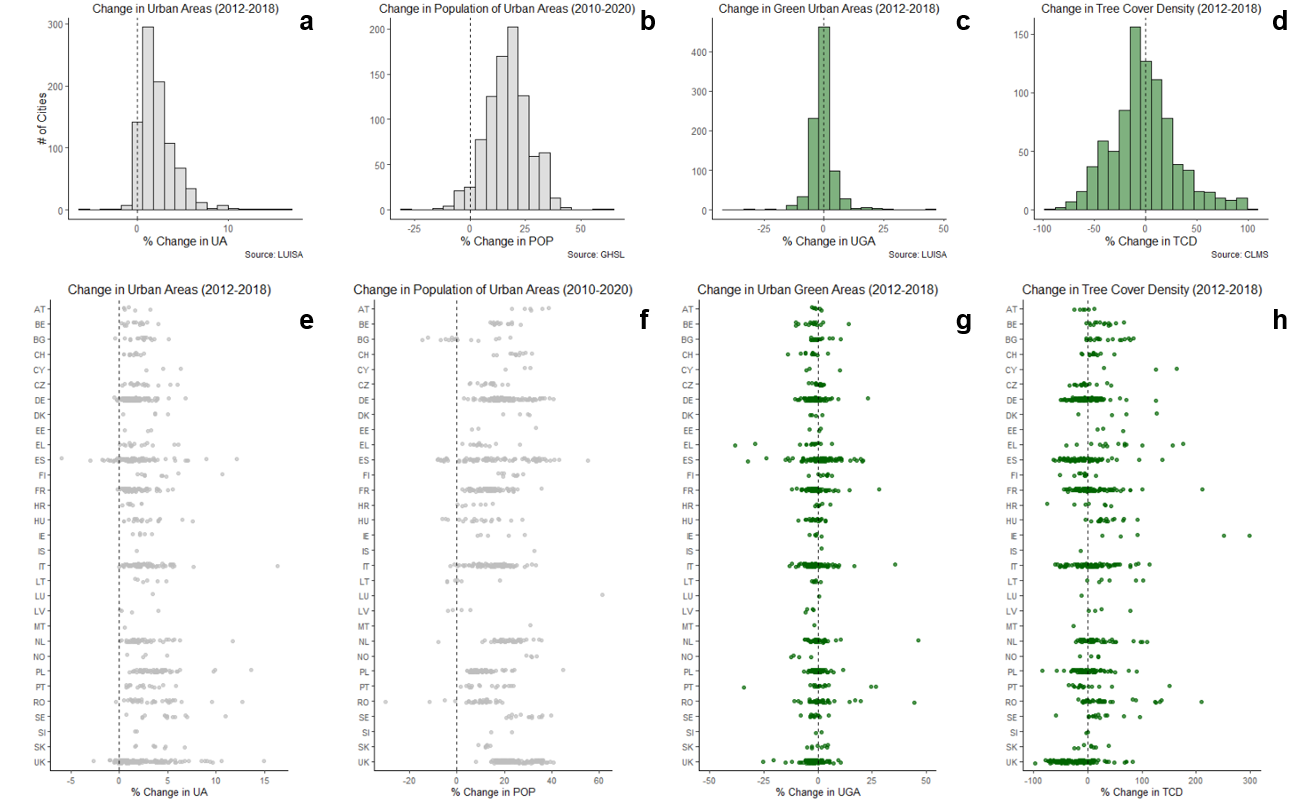


**Supplementary Figure 3:** Comparison between the change (a)-(e) urban area (b)-(f) population in urban area (c)-(g) green urban area (d)-(h) tree cover density during the last decade (2012-2018, or 2010-2020) at city level (upper panels) and their aggregation in histograms (lower panels). The vertical dashed line represents the zero value, thus all the values above zero correspond to an increase of the specific variable

**
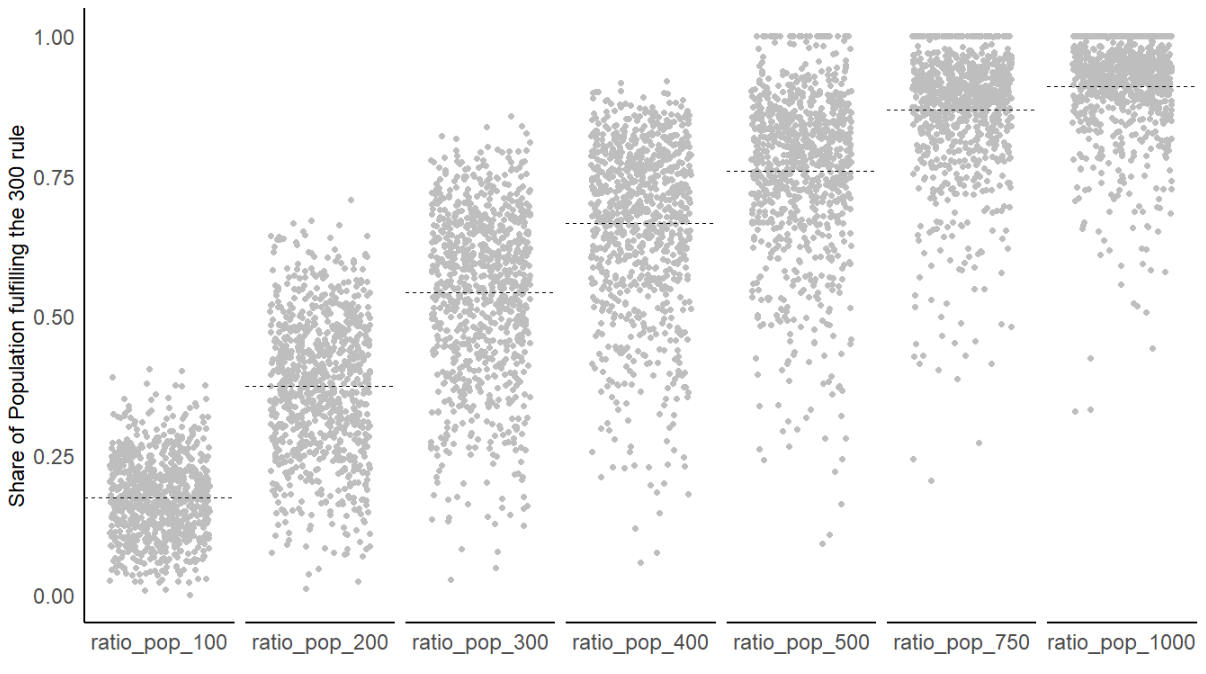
**

**Supplementary Figure 4:** Percentage of European cities population living within specified distance to parks or green spaces. Grey dots show the individual trend for each of the 862 cities in the dataset, while the black dashed line indicates the median.

**
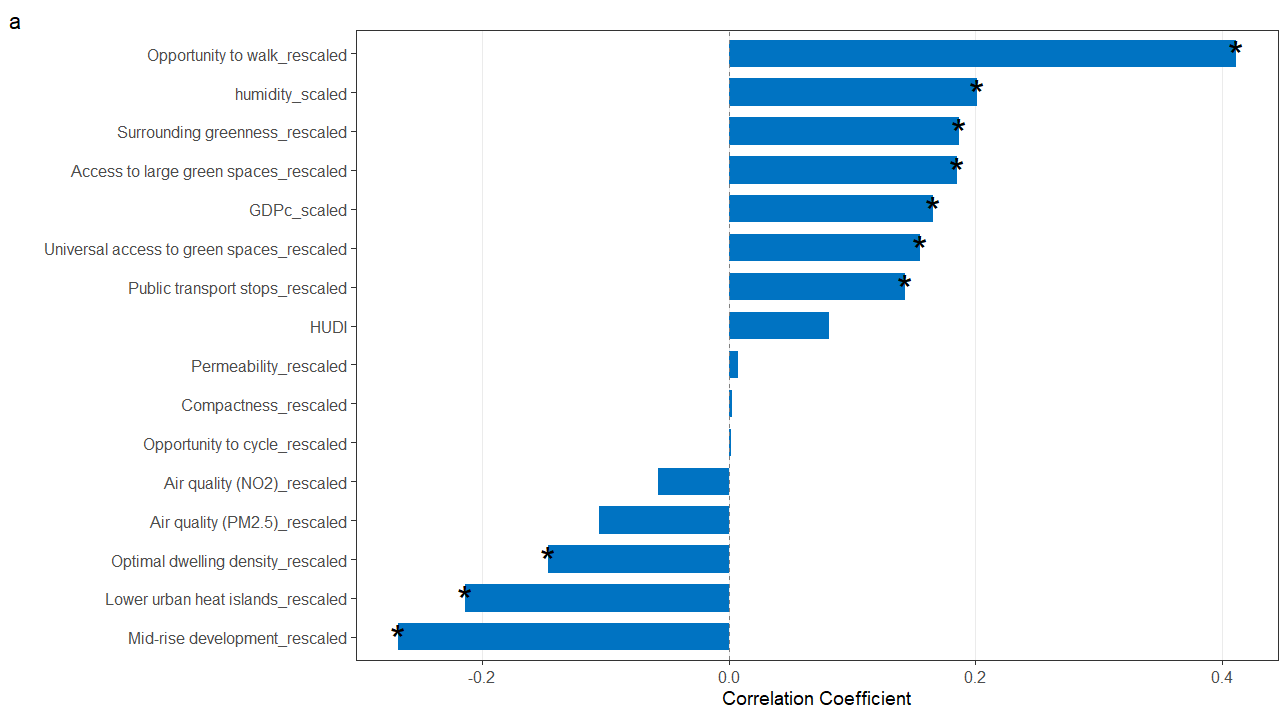
**

**
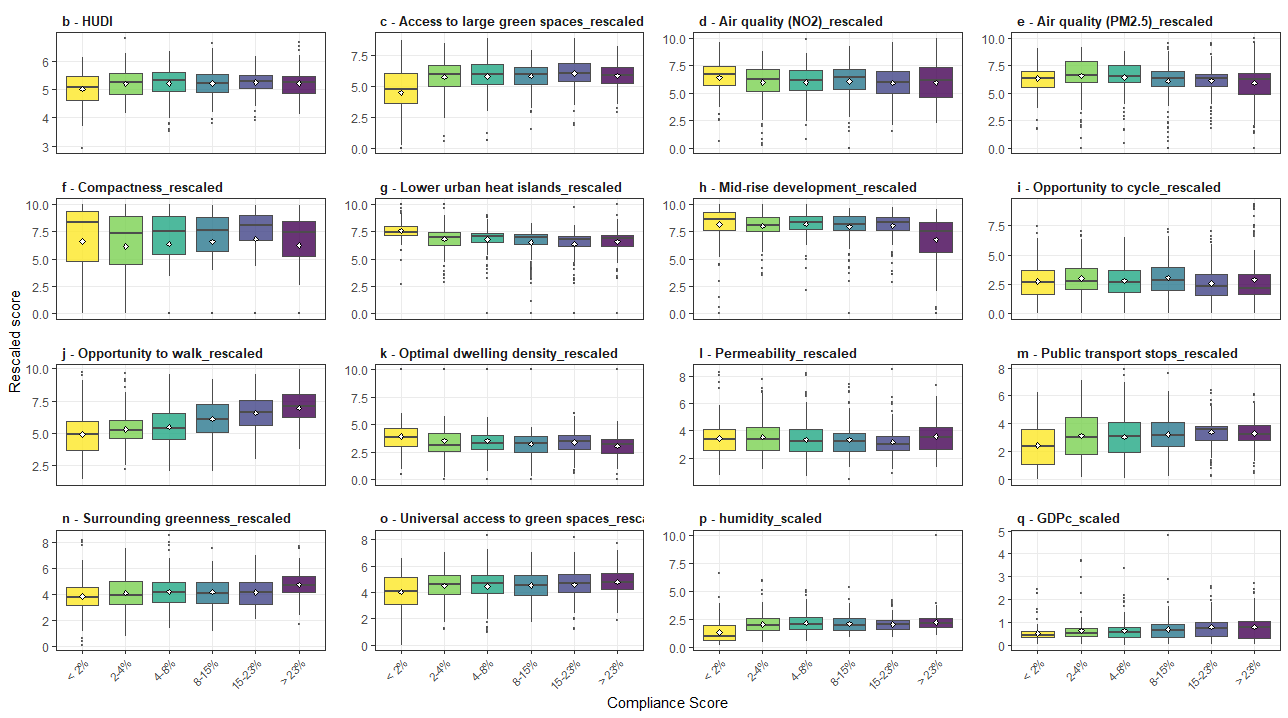
**

**Supplementary Figure 5:** Correlation between the percentage of population in European cities meeting the 3-30-300 rule (a) and 16 indicators across four overarching domains of urban design, sustainable transportation, environmental quality, and green space accessibility (b)-(q). Note that 14 out of the 16 indicators are obtained from Montana et al., [1], while the values of Aridity Index and GDPc are calculated as per Figure 3. The population meeting the 3-30-300 rule have been divided in 6 clusters for better visualization, while the correlation on the right panels have been conducted across all the values not separated in clusters. The barplots show the correlation coefficient and they also report the p-value of such correlations. We added a star when the correlation is significant with a p-value < 0.0001.


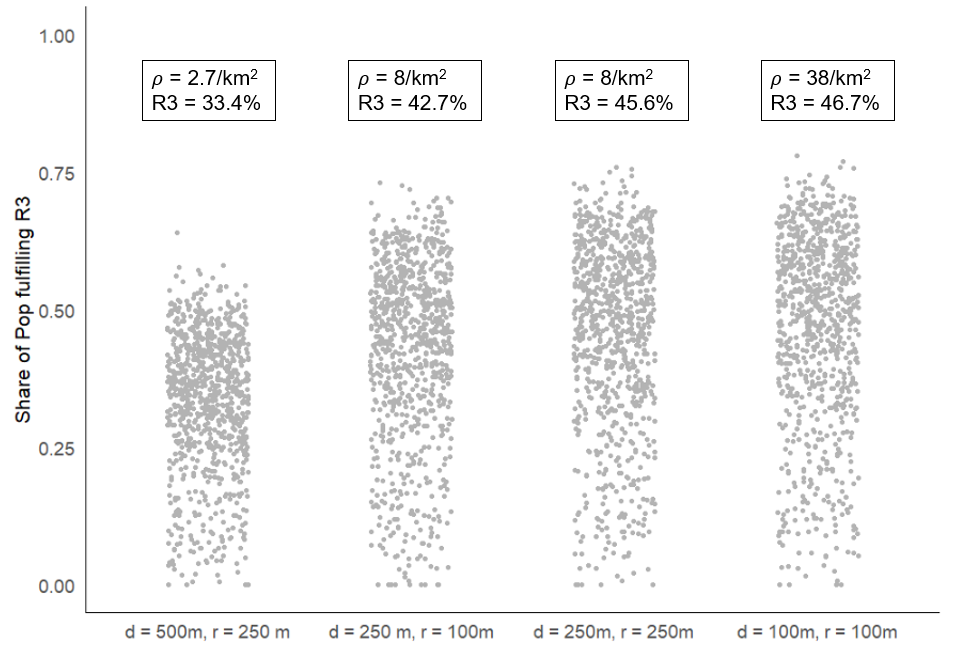


**Supplementary Figure 6**: The scatter plot illustrates the range of estimated population shares meeting the Rule R3 criteria (visibility of at least 3 trees) across 862 European cities, based on four distinct parameter sets for the viewshed analysis. The analysis varied the minimum distance between viewpoints (d: 100m, 250m, 500m) and the viewshed radius (r: 100m, 250m). The final parameter set chosen for the main text results - d = 100m (highest viewpoint density) and r = 100m (most refined spatial resolution) - yielded the maximum population share meeting the criteria, with an estimated 46.7% of the population having visibility of at least three trees. This selection offers the highest feasible spatial resolution, constrained by the input dataset limitations.


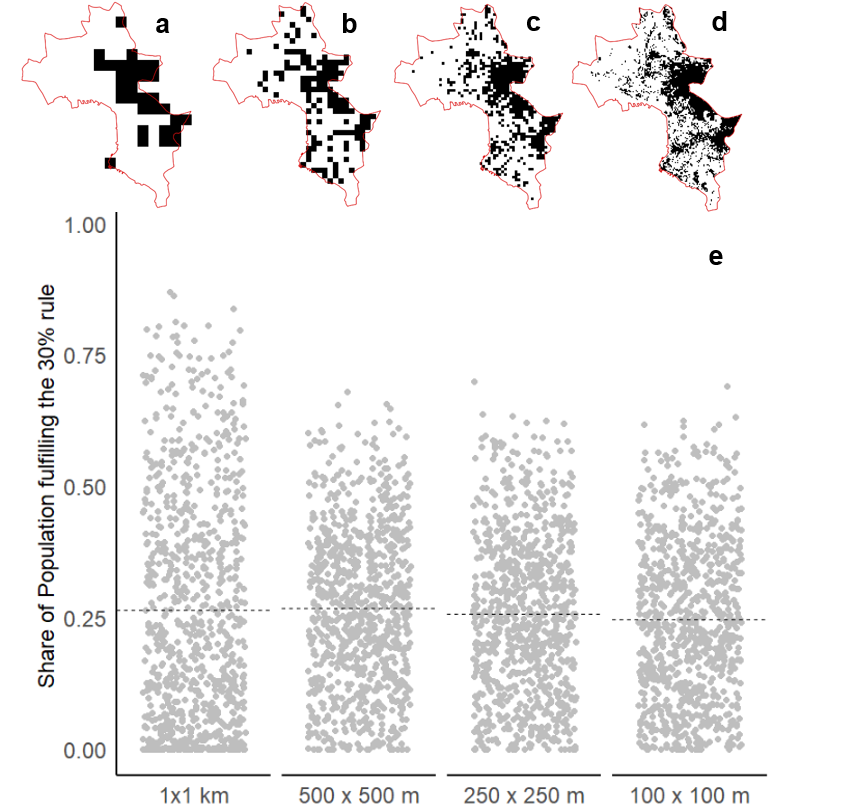


**Supplementary Figure 7:** sensitivity analysis of 30% tree cover compliance across grid scales. Panels (a–d): Visual representation of neighborhood scaling using Thessaloniki, Greece, as a case study. Black pixels indicate "neighborhoods" that meet or exceed the 30% tree cover threshold. The spatial resolution increases from (a) to (b), representing a decrease in grid cell size from 1x1 km to 0.1x0.1 km. In Panel (e) we reported the sensitivity of population compliance to neighborhood size. Each data point represents a specific city from the database, positioned according to the grid scales shown in panels (a–d). The black horizontal dashed lines represent the mean compliance rate across all cities at each respective scale.


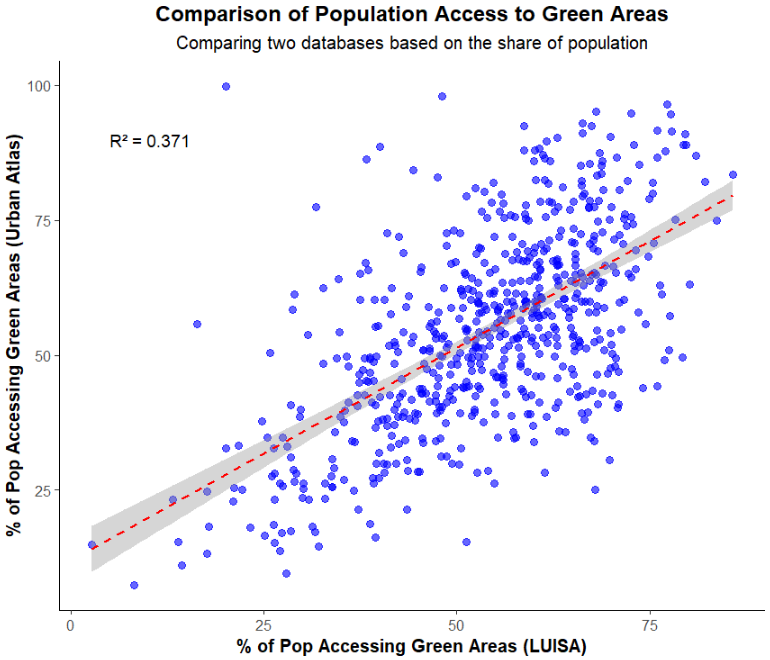


**Supplementary Figure 8:** Comparison between the share of European cities' population meeting the R-300 rule with the proportion of urban center populations having access to at least 1 hectare of green urban areas within a 400-meter walk, as reported in the DG REGIO study.


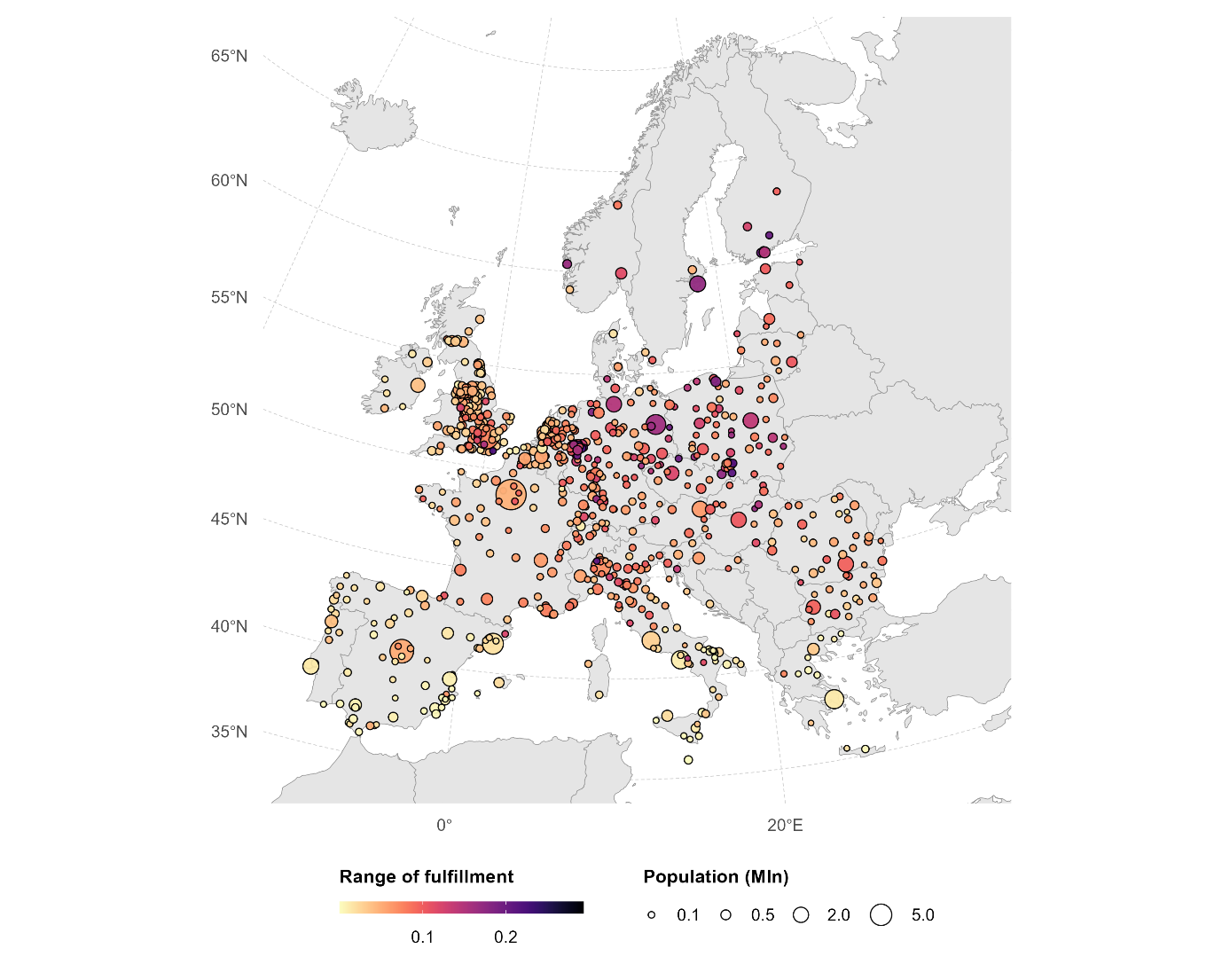
**Supplementary Figure 9:** Spatial representation of the variability in adherence to the 3-30-300 guideline across 862 European cities. The range depicted is derived from the difference between the maximum and minimum fulfillment values of the rule across 16 distinct scenarios from the sensitivity analysis. Circle sizes are scaled proportionally to each city's total population


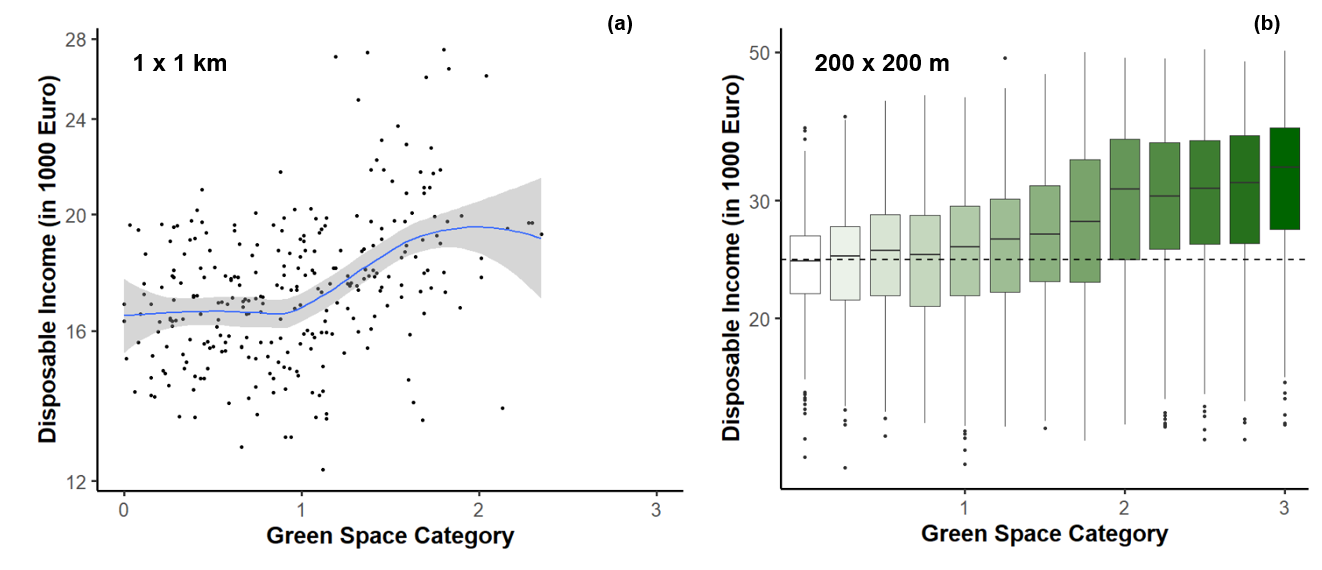


**Supplementary Figure 10:** Comparative analysis of two datasets on the relationship between disposable income and green space abundance in Lyon (France). The left panel uses coarser data (1 x 1 km) on disposable income from Mikou et al. [2], while the right panel employs the FiLoSoFi (INSEE) database [3], offering finer resolution data (200 x 200 m) on residents' income in France.


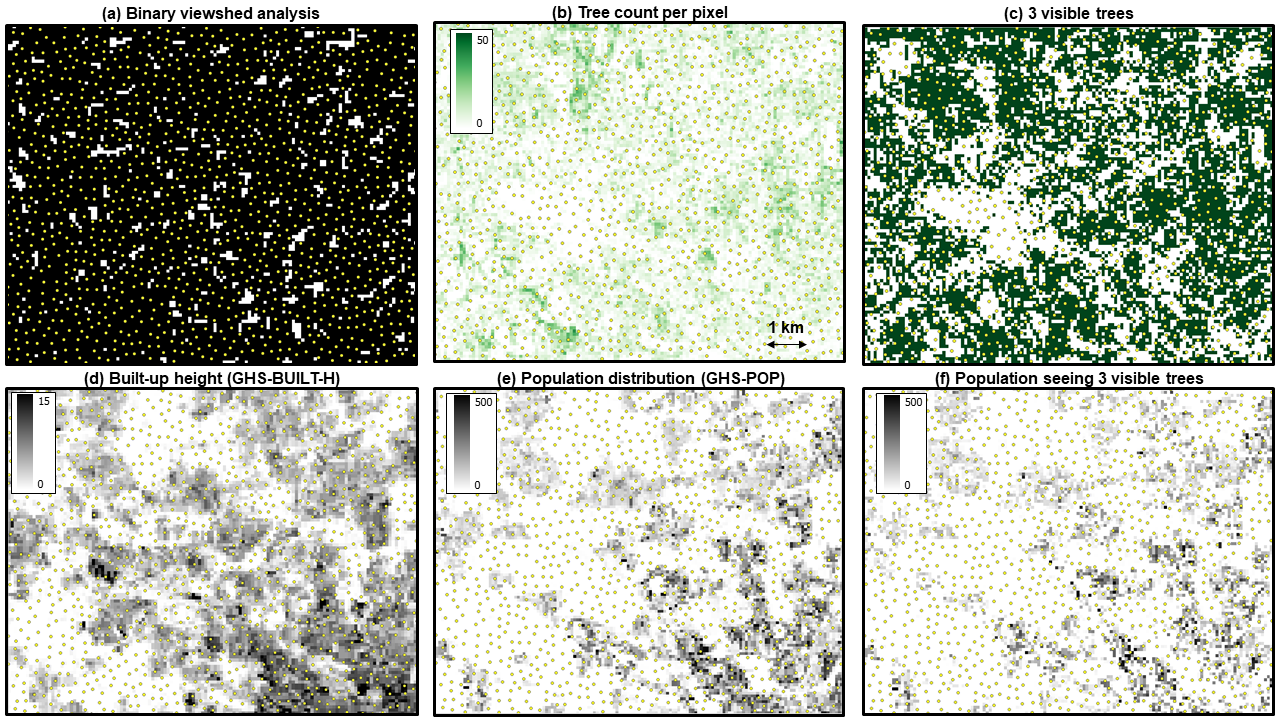


**Supplementary Figure 11:** Example of results extraction for Rule 3 with a minimum distance between points of 100 meters and an analysis radius of 100 meters. Panel (A) shows the binary viewshed analysis, where black pixels indicate visible areas from the yellow viewpoints. White areas are not visible due to obstacles provided in the Built-up height layer (Panel D). Panel (B) displays the number of trees identified per pixel. In this panel, we isolated pixels with at least three trees and combined this new binary layer with Panel (A). From these two rasters, we retained only the pixels with a value of 2, indicating that they contain at least three trees and are within visible areas (Panel C). This raster is then intersected with the population distribution obtained from the Global Human Settlement Layer (GHS-POP, Panel E), to obtain the final dataset of population seeing at least three visible trees (Panel F). All the analyses are conducted on rasters with 100 m of spatial resolution.

# **Supplementary Tables**

**Supplementary Table 1:** Summary of the databases used in the assessment of the 3-30-300 rule.

| **Dataset** | **Rule** | **Year** | **Details** | **Reference** |
| --- | --- | --- | --- | --- |
| PlanetScope | 3, 30 | 2019 | Individual tree points derived from 3 m spatial resolution tree cover products (gaussian heatmaps). | 27-28 |
| GHSL-BUILT-H | 3 | 2018 | The spatial raster dataset depicts the spatial distribution of the building heights per cell. Resolution of 100 x 100 m | 30 |
| LUISA | 300 | 2018 | The LUISA Base Map 2018 is a high-resolution land use/land cover map (50 x 50 m). We considered only the urban areas pixels where we have information about green urban vegetation (i.e. parks) | 25 |
| GHSL-POP | 3, 30, 300 | 2020 | The spatial raster dataset depicts the distribution of population, expressed as the number of people per cell (100 x 100 m) | 29 |
| Urban Audit | 3, 30, 300 | 2020 & 2021 | Extent of 862 EU (2021) and UK (2020) cities. We choose only the City Core and not the larger Functional Urban Area | 61 |

**Supplementary Table 2**: Results of the sensitivity analysis examining the proportion of the European cities' population that meets the 3-30-300 rule, focusing on the R3 and R30% parameters. Here, D denotes the minimum distance between viewpoints, while r represents the radius used in the viewshed analysis. The rows of the table correspond to varying grid sizes, which delineate the spatial definition of city neighborhoods.

| **R3→**  **↓R30** | **D = 500,**  **r = 250** | **D = 250,**  **r = 250** | **D = 250,**  **r = 100** | **D = 100,**  **r = 100** |
| --- | --- | --- | --- | --- |
| 100 x 100 m | 9.5% | 13.5% | 12.1% | 13.3% |
| 250 x 250 m | 9.4% | 12.9% | 11.8% | 13.0% |
| 500 x 500 m | 9.3% | 12.6% | 11.7% | 12.9% |
| 1000 x 1000 m | 9.8 % | 13.2% | 12.3% | 13.5% |

**Supplementary Table 3:** Comparison of pan-European 3-tree rule estimates with high-resolution local case studies. This table benchmarks our 100-m resolution continental findings against localized studies utilizing building-level data. Despite the coarser resolution of the GHS-BUILT-H and GHS-POP layers, our results remain broadly consistent with local assessments. Discrepancies are primarily attributed to the metric used (share of population vs. share of buildings) and the neighborhood-level averaging inherent in 100-m grain analysis.

| **City** | **Reference** | **Local Study (High-Res)** | **This Study (100m)** | **Methodological Difference** |
| --- | --- | --- | --- | --- |
| **Barcelona (ES)** | Nieuwenhuijsen et al. [4] | 43% | **31%** | Local study used individual surveys/perception. |
| **Amsterdam (NL)** | Coreser et al. [5] | 50% | **46%** | Local study: % of Buildings; Our study: % of Population. |
| **Aix-en-Provence (FR)** | Antonio Lopez et al. [6] | 68% | **62%** | Local study: % of Buildings; Our study: % of Population. |
| **Florence (IT)** | Antonio Lopez et al. [6] | 38% | **59%** | Local study: % of Buildings; Our study: % of Population. |
| **Turin (IT)** | Battisti et al. [7] | 40% | **48%** | Local study: Neighborhood-level average. |

# **Supplementary References**

1. Montana, F., Mueller, N., Barboza, E. P., Khomenko, S., Iungman, T., Cirach, M., ... & Nieuwenhuijsen, M. (2025). Building a Healthy Urban Design Index (HUDI): how to promote health and sustainability in European cities. The Lancet Planetary Health, 9(6), e511-e526.
2. Mikou, M., Vallet, A., Guivarch, C., & Makowski, D. (2025). High‐resolution downscaling of disposable income in Europe using open‐source data. Earth's Future, 13(1), e2024EF004576.
3. 44. National Institute of Statistics and Economic Studies. (2019). Social and fiscal localized income, 2019: Filoso 2019. Retrieved from [<https://www.insee.fr/fr/statistiques/7655515>]
4. Nieuwenhuijsen, M. J., Dadvand, P., Márquez, S., Bartoll, X., Barboza, E. P., Cirach, M., ... & Zijlema, W. L. (2022). The evaluation of the 3-30-300 green space rule and mental health. Environmental research, 215, 114387.
5. Croeser, T., Sharma, R., Weisser, W. W., & Bekessy, S. A. (2024). Acute canopy deficits in global cities exposed by the 3-30-300 benchmark for urban nature. Nature Communications, 15(1), 9333.
6. Lopez, M. A., De Marco, A., Anav, A., Sorrentino, B., Paoletti, E., Manzini, J., ... & Sicard, P. (2025). The 3–30–300 rule Compliance: A geospatial tool for urban planning. Landscape and Urban Planning, 261, 105396.
7. Battisti, L., Giacco, G., Moraca, M., Pettenati, G., Dansero, E., & Larcher, F. (2024). Spatializing Urban Forests as Nature-based Solutions: a methodological proposal. Cities, 144, 104629.
